# SSTR-2 as a potential tumour-specific marker for fluorescence-guided meningioma surgery

**DOI:** 10.1007/s00701-018-3575-z

**Published:** 2018-06-01

**Authors:** B. M. Dijkstra, A. Motekallemi, W. F. A. den Dunnen, J. R. Jeltema, G. M. van Dam, F. A. E. Kruyt, R. J. M. Groen

**Affiliations:** 1 Department of Neurosurgery, University Medical Center Groningen, University of Groningen, Hanzeplein 1, P.O. Box 30.001, 9700 RB Groningen, The Netherlands; 2 Department of Neurosurgery, University Medical Center Münster, Münster, Germany; 3 Department of Pathology, University Medical Center Groningen, University of Groningen, Groningen, The Netherlands; 4 Department of Surgery, Nuclear Medicine and Molecular Imaging and Intensive Care, University Medical Center Groningen, University of Groningen, Groningen, The Netherlands; 5 Department of Medical Oncology, University Medical Center Groningen, University of Groningen, Groningen, The Netherlands

**Keywords:** Intracranial meningioma, Fluorescence guided surgery, Intraoperative imaging, Somatostatin receptor subtype 2, Biomarker

## Abstract

**Background:**

Meningiomas are the most frequently occurring primary intracranial tumours in adults. Surgical removal can only be curative by complete resection; however surgical access can be challenging due to anatomical localization and local invasion of bone and soft tissues. Several intraoperative techniques have been tried to improve surgical resection, including intraoperative fluorescence guided imaging; however, no meningioma-specific (fluorescent) targeting has been developed yet. Here, we aimed to identify the most promising biomarkers for targeted intra-operative fluorescence guided meningioma surgery.

**Methods:**

One hundred forty-eight meningioma specimens representing all meningioma grades were analysed using immunohistochemistry (IHC) on tissue microarrays (TMAs) to determine expression patterns of meningioma biomarkers epithelial membrane antigen (EMA), platelet-derived growth factor β (PDGF-β), vascular endothelial growth factor α (VEGF-α), and somatostatin receptor type 2 (SSTR-2). Subsequently, the most promising biomarker was selected based on TArget Selection Criteria (TASC). Marker expression was examined by IHC in 3D cell culture models generated from freshly resected tumour material.

**Results:**

TMA-IHC showed strongest staining for SSTR-2. All cases were positive, with 51.4% strong/diffuse, 30.4% moderate/diffuse and only 18.2% focal/weak staining patterns. All tested biomarkers showed at least weak positivity in all meningiomas, regardless of WHO grade. TASC analysis showed that SSTR-2 was the most promising target for fluorescence guided imaging, with a total score of 21 (out of 22). SSTR-2 expression was determined on original patient tumours and 3D cultures of three established cultures.

**Conclusions:**

SSTR-2 expression was highly sensitive and specific in all 148 meningiomas, regardless of WHO grade. According to TASC analysis, SSTR-2 is the most promising receptor for meningioma targeting. After establishing in vitro meningioma models, SSTR-2 cell membrane expression was confirmed in two of three meningioma cultures as well. This indicates that specific fluorescence in an experimental setting can be performed for the further development of targeted fluorescence guided meningioma surgery and near-infrared fluorescent tracers targeting SSTR-2.

## Introduction

Meningiomas are the most frequently occurring intracranial tumours in adults, accounting for approximately one third of cases [37]. They are classified by the World Health Organization (WHO) into three malignancy grades with 15 histologic subtypes [26]. Treatment is usually only curative with complete surgical resection [6, 50] and aims for both complete tumour removal and preservation of neurological function [29, 55]. Although mostly benign and slow growing, all meningiomas can pose surgical challenges due to anatomical localization and local invasion of bone and soft tissues, leading to residual tumour tissue. Incomplete resection is one of the risk factors for recurrence [6].

One of the proposed techniques to facilitate resection is intraoperative fluorescence-guided imaging. Several fluorescent dyes (5-aminolevulinic acid, fluorescein, and indocyanine green) have been tried [1, 10, 14, 25, 33]; however, evidence regarding the benefit of applying these dyes is unavailable. Furthermore, fluorescent dyes currently lack specificity. The concept of targeted fluorescence is appealing, due to high sensitivity and specificity rates. Identification of meningioma-specific biomarkers is a first and essential step in this concept. Comparable targeted fluorescent techniques have been established in other tumour types, e.g. in ovarian carcinoma [17, 53] and peritoneal metastases of colorectal carcinomas [18] targeting αvβ3-integrin or folate receptor α and VEGF-α, respectively. For the development of a similar approach in meningioma surgery, various biomarkers have been suggested, including epithelial membrane antigen (EMA), platelet-derived growth factor beta (PDGF-β), vascular endothelial growth factor A (VEGF-α), and somatostatin receptor type 2 (SSTR-2) [3, 5, 9, 11, 12, 16, 19, 21, 28, 30, 32, 38, 39, 43–45, 48, 52, 56]. However, the suitability of these markers for fluorescence-guided imaging meningioma surgery has not yet been investigated.

In this study, we aimed to make a step-wise approach: (1) identifying meningioma-specific candidate biomarkers (EMA, PDGF-β, VEGF-α and SSTR-2); (2) selecting the most promising tumour-specific marker; and (3) confirming its expression in in vitro cultures derived from fresh meningioma specimens.

## Methods and materials

### Part 1: identifying meningioma-specific candidate biomarkers

Specimens of previously untreated, primary intracranial meningiomas resected between January 2006 and December 2010 were retrospectively analysed for the expression of four potential biomarkers (i.e. EMA, PDGF-β, VEGF-α and SSTR-2). A total number of 148 meningioma specimens were available for analysis. All patient data were anonymized according to the regulations of the Medical Ethical Research Committee of the University Medical Center Groningen.

Meningioma samples were examined using tissue microarrays (TMAs). TMA sections were deparaffinised with xylene, rehydrated in ethanol, and rinsed in distilled water. After antigen retrieval with a Tris-EDTA or Tris-HCl buffer, endogenous peroxidase was blocked for 30 min using a 0.1% H_2_O_2_ PBS solution. Respective antibody staining was performed at room temperature. The selected primary antibodies of interest were anti-EMA (mouse monoclonal, Dako), anti-PDGFR-β (rabbit polyclonal, Santa Cruz), anti-VEGF-α (rabbit polyclonal, Santa Cruz) and anti-SSTR-2 (rabbit monoclonal, Epitomics). We used normal cerebellar and anterior pituitary tissue as positive controls. Additionally, we omitted the primary antibody and used IgG controls. Secondary and tertiary antibody staining was performed for 30 min. All sections were subjected to a 3,3-diaminobenzidine solution for 10 min and finally counterstained with haematoxylin for 2 min, dehydrated in ethanol, cleared, mounted and cover slipped.

For immunohistochemical (IHC) evaluation, TMA sections were scanned with an ultra-resolution digital scanner ScanScope CS®, Aperio® with × 20 image magnification and evaluated with Aperio ImageScope® software. Each tissue core of the TMA section was scored using the following scoring method: negative, (0); weak/focal staining, (1); moderate/diffuse staining, (2); strong/diffuse staining, (3). Two authors (AM and WFD) independently evaluated tissue cores and in case of discrepant scores, consensus was reached by way of discussion between both evaluators. Cores were regarded as non-informative and consequently dismissed when > 50% tissue was lost or presented inappropriate amounts of collagen staining. Tumour specimens which were represented by less than two complete cores were excluded. A mean score was calculated for each specimen and specimens with an average score of 1 were considered positive, whereas specimens with a mean score of ≥ 2 were summarized as “high score”.

#### Statistical analysis

All statistical analyses were performed using IBM® SPSS® Statistics 20. Spearman rank-order correlation was used to find correlation between targets and WHO classifications. All reported *P* values were two sided and a value of *P* ≤ 0.05 was considered as statistically significant.

### Part 2: selecting the most promising tumour-specific marker

To investigate the usefulness of these markers for intra-operative fluorescence-guided imaging, the TArget Selection Criteria (TASC) [54] were utilized for the selection of the most promising targets, as depicted in Table 1.

**Table 1:** TASC scoring system

Criteria	Characteristics		Score
I	Extracellular protein localization	Bound to cell surface (receptor)	5
		In close proximity of tumour cell	3
II	Diffuse upregulation through tumour tissue		4
III	Tumour-to-normal ratio > 10		3
IV	Percentage upregulation in patients	> 90%	6
		70–89%	5
		50–69%	3
		10–49%	0
V	Previously successfully imaged in vivo		2
VI	Enzymatic activity		1
VII	Internalization		1
Maximum: 22			
Potential target ≥ 18			

### Part 3: confirming expression in in vitro cultures

Meningioma 3D cell culture models were established to provide an accurate model for the disease [13, 24]. Surgical leftover fresh tumour tissue was washed with ice-cold PBS and mechanically dissociated. After adding 15 to 20 ml accutase, tissue incubated for 30 min at room temperature. The suspension was passed through a 70-μm cell strainer to procure single cells and pelleted. Cells were seeded in T75 flasks with medium containing DMEM/F12 supplemented with 2% B27, 20 ng/ml EGF, 20 ng/ml bFGF [22], and 2% pen/strep.

For IHC analyses, 3D cultures were dissociated with accutase and cytospun. Subsequently, cells were fixated with 4% formaldehyde, washed with PBS and underwent a blocking step with 1% H_2_O_2_ in PBS for 10 min. Cells were then incubated with anti-SSTR-2 (1:100; MAB4224, R&D systems) for 1 h, followed by incubation of the corresponding secondary and tertiary antibodies diluted at 1:50 in PBS with 1% BSA and 1% AB serum for 30 min. Lastly, cells were incubated with 5% 3-amino-9-ethylcarbazole diluted in acetate buffer with 0.1% hydrogen peroxide for 10 min, counterstained with haematoxylin for 2 min, and mounted with Kaiser’s glycerin for microscopic examination using a Leica DM 3000 microscope.

## Results

### Part 1: identifying meningioma-specific candidate biomarkers

#### Patient characteristics

Patient and tumour characteristics are summarized in Table 2. The age at surgery ranged from 4 to 79 years. Tumour specimens were obtained from 52 male and 96 female patients. Our study revealed 124 WHO I (83.8%), 22 WHO II (14.9%) and 2 WHO III (1.4%) meningiomas. All tested biomarkers showed at least weak positivity in all meningiomas, regardless of WHO grade. No association was found between WHO grade and the expression rates of the potential targets using Spearman rank order correlation (Table 3).

**Table 2:** Patient and tumour specimen characteristics

	Men	Women
Percent (*n* (%))	52 (35.1)	96 (64.9)
Age (years) (mean (range))	47.2 (4.4–79.8)	52.4 (4.2–77.5)
Meningioma grade (*n* (%*))		
WHO I	38 (25.7)	86 (58.1)
WHO II	12 (8.1)	10 (6.8)
WHO III	2 (1.4)	0 (0.0)

*Percentage of all meningiomas

**Table 3:** Correlation between target expression and WHO grades

Target	Spearman correlation (*p* value)
EMA	.498
VEGF-α	.909
PDGFR-β	.255
SSTR-2	.647

#### Target expression in TMA sections

Both cerebellar and anterior pituitary tissue showed SSTR-2-positivity. Additionally, white matter in the cerebellar tissue was SSTR-2 negative, as expected. Omission of the primary antibody revealed no SSTR-2 staining and aspecific binding with IgG was not observed.

**Fig. 1:**
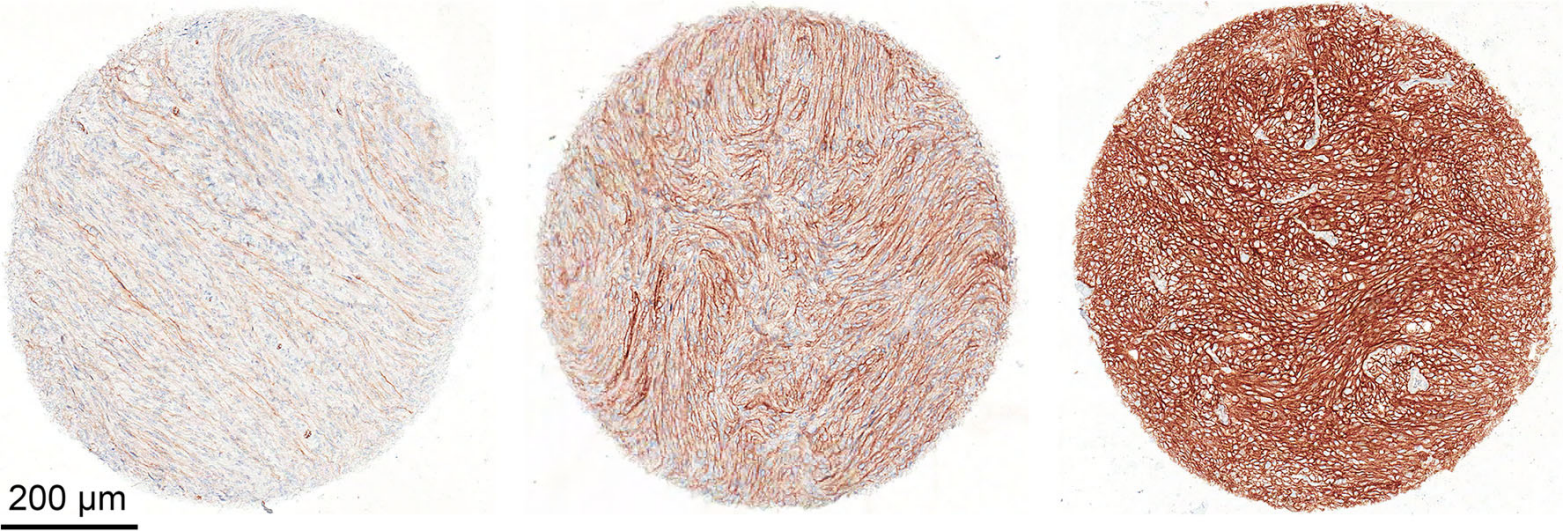
Representative images of SSTR-2 stained TMA-IHC cores and scoring approach. Shown are weak/focal (left), moderate/diffuse (middle), and strong/diffuse (right) staining patterns

TMA-IHC results are summarized in Table 4. Of all TMA meningioma specimens (588), 99.8% was IHC-positive for the investigated targets with at least two or more valid tissue cores. The number of non-informative/invalid cores was low for PDGFR-β (0.7%) and VEGF-α (2.0%) and all cores for EMA and SSTR-2 were valid. The IHC staining score for SSTR-2 was the most robust, resulting in positivity for SSTR-2 in all specimen: moderate/diffuse or strong/diffuse positivity in 81.8% (“high score”) and focal/weak positivity in only 18.2% of all cases. Representative examples illustrating SSTR-2 expression are shown in Fig. 1.

**Table 4:** Summary of IHC results

Target	Valid cores	Weak/focal (%)	Moderate/ diffuse (%)	Strong/diffuse (%)	High score* (%)
EMA	148	16 (10.8)	113 (76.4)	19 (12.8)	132 (89.2)
VEGF-α	145	45 (31.0)	93 (64.1)	7 (4.8)	100 (69.0)
PDGFR-β	147	34 (23.1)	107 (72.8)	6 (4.1)	113 (76.9)
SSTR-2	148	27 (18.2)	45 (30.4)	76 (51.4)	121 (81.8)

Shown percentages are valid ratios for the respective target

*Defined as moderate/diffuse or strong/diffuse staining

### Part 2: selecting the most promising tumour-specific marker

The selected markers were evaluated using TASC based on IHC-TMA results (Table 5). Using these criteria, SSTR-2 is the most promising target for intra-operative use with a total TASC score of 21. In addition, EMA, PDGFR-β and VEGF-α seem to be high potential targets with TASC scores of 20, 20 and 18, respectively.

**Table 5:** Target selection by applying TASC

TASC item	I Localization	II Expression pattern* ^≠^	III T/N ratio	IV Upregulation in patients* (%)	V In vivo imaging	VI Enzymatic activity	VII Internalization	Score
Target								
EMA	Transmembrane	Diffuse	High	100	Yes [34, 49]	ND	ND	20
VEGF-α	Secreted	Diffuse	High	100	Yes [35, 36, 47]	ND	ND	18
PDGFR-β	Transmembrane	Diffuse	High	100	Yes [15]	ND	ND	20
SSTR-2	Transmembrane	Diffuse	High	100	Yes [21, 23, 52]	Yes [46]	ND	21

*ND*, not described; *T/N ratio*, tumour-to-normal tissue ratio

*Results based on this study

^≠^Expression patterns are considered “diffuse”, if moderate/diffuse or strong/diffuse staining is present in more than 66% of included cases

### Part 3: confirming expression in in vitro cultures

#### Generating meningioma cultures

**Fig. 2:**
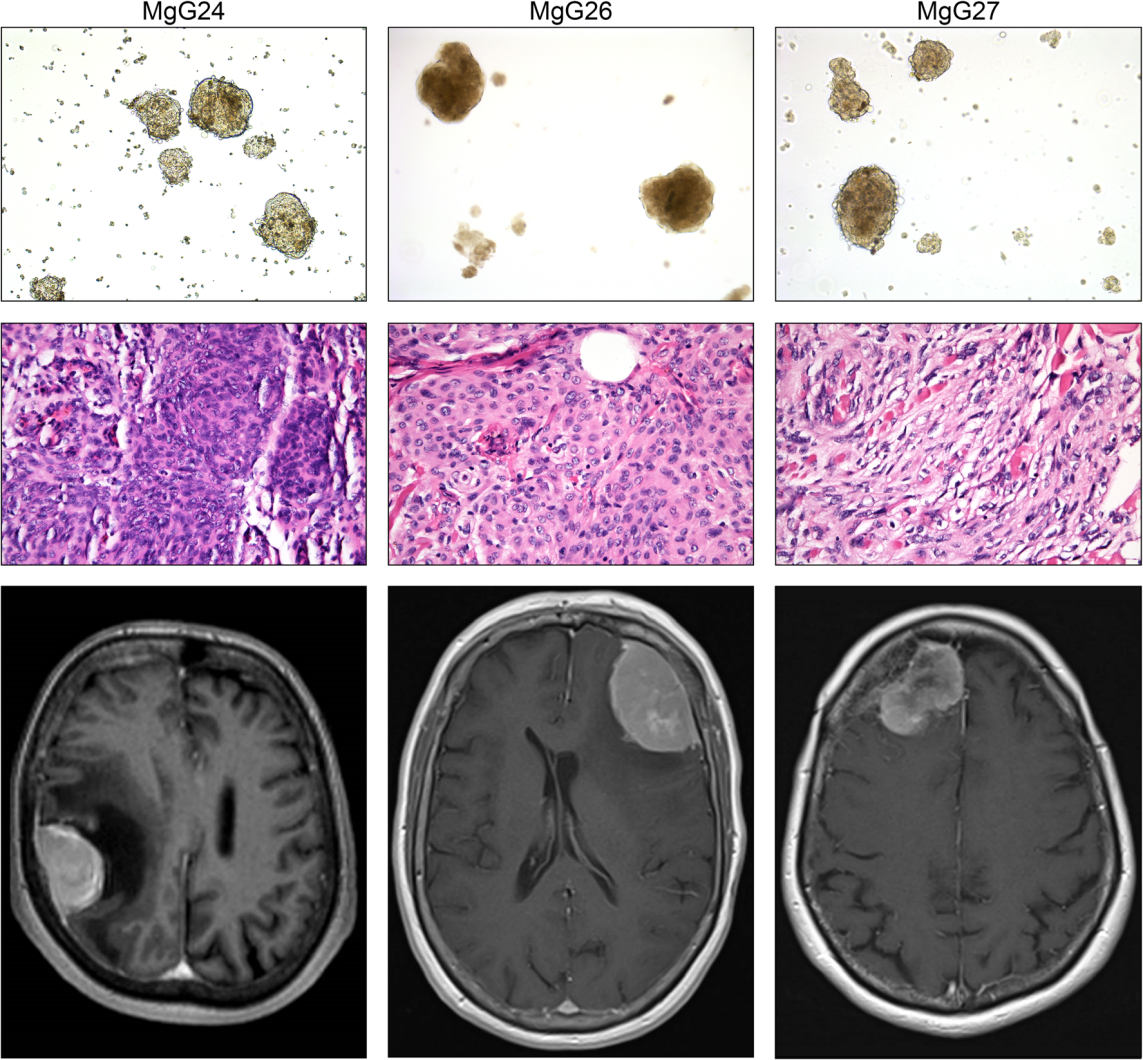
Top panel shows micrographs of 3D meningioma cultures with × 10 magnification. Middle panel depicts micrographs of H&E stained original patient tumour at × 40 magnification. Bottom panel are gadolinium-enhanced MRI scans. 3D cultures showed aggregated cell formation into a sphere. H&E stained tumour samples confirmed the diagnosis of meningioma in all three cases. MRI scans revealed meningiomas at the convexity and skull base

In vitro cultures were established to further explore the potential of SSTR-2 as a meningioma-specific marker in a translational model. After processing the freshly resected material, 11 of 22 cultures (50%) generated 3D cultures after 7 days (Fig. 2, top panel). However, growth decreased after three to four passages. A selection of three cultures named MgG24, MgG26 and MgG27 was characterized in more detail. These cultures originated from three female patients with a mean age of 65.7 years (range 60–77; SD 9.8). All meningiomas were WHO grade I, with one transitional and two meningothelial meningiomas (Fig. 2, middle panel). Two meningiomas were located at the convexity and one at the skull base (Fig. 2, bottom panel).

#### SSTR-2 expression in in vitro meningioma cultures

**Fig. 3:**
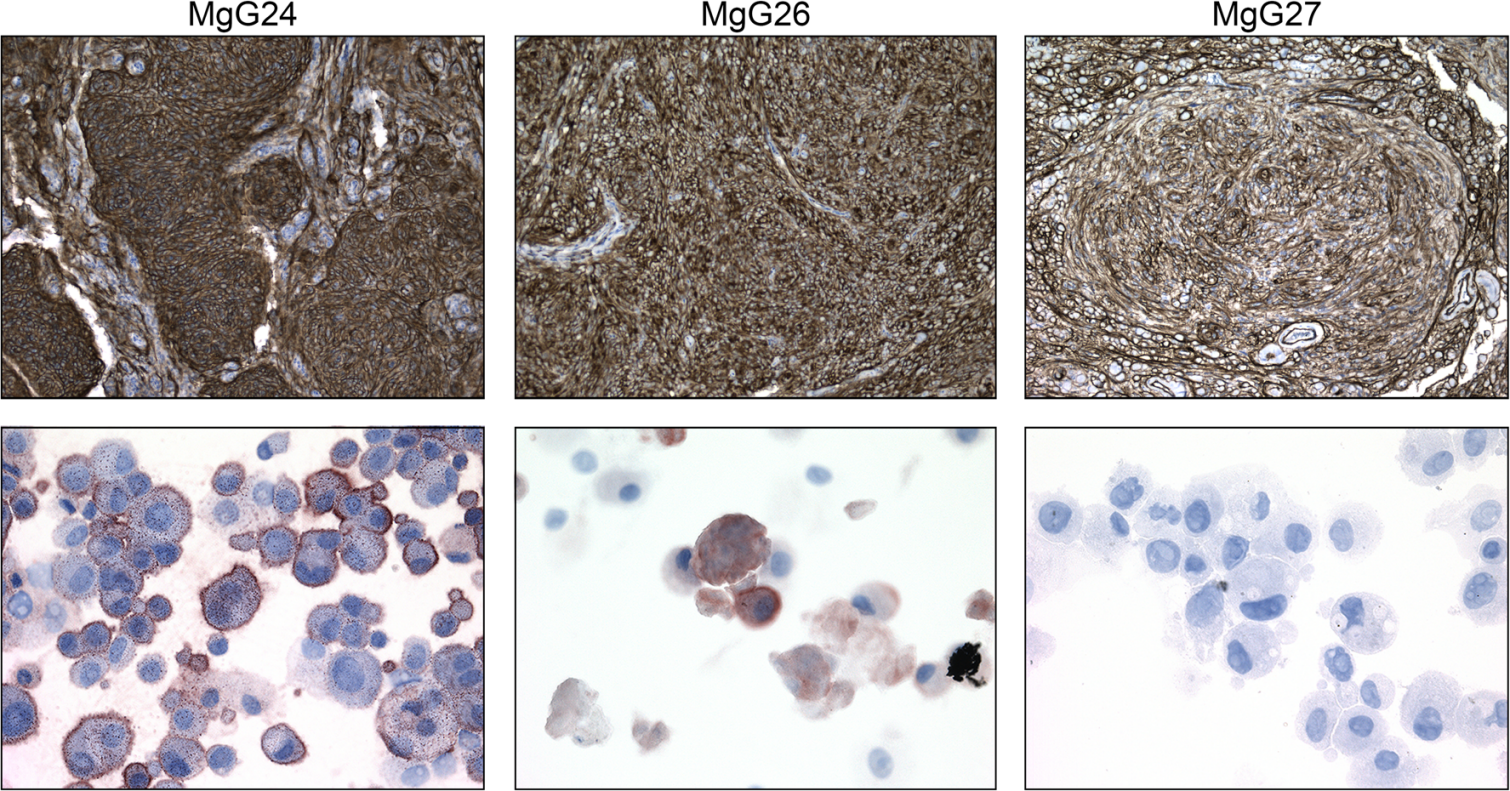
Top panel depicts micrographs from primary tumour stained for SSTR2 at × 10 magnification. Bottom panel shows micrographs of dissociated cells cultured as 3D and stained for SSTR2 at × 40 magnification. Patient material showed SSTR-2 membrane staining in all tumours. Two of three dissociated 3D cultures revealed SSTR-2 positive membranous staining in a fraction of cells

SSTR-2 expression was determined on original patient tumours and 3D cultures of the three established cultures. All patient tumours were strongly positive for SSTR-2 at the cell membrane (Fig. 3, top panel). However, of the dissociated 3D cultures, only MgG24 and MgG26 are SSTR-2 positive with a cell membranous staining. It should be noted that not all cells are (equally) positive in both cultures: some cells show no or weak SSTR-2 positivity, whereas in other cells, the staining is strongly positive (Fig. 3, bottom panel).

## Discussion

The first aim of this study was to identify the expression pattern of a series of preselected markers (EMA, PDGF-β, VEGF-α and SSTR-2) in meningiomas using TMA-IHC, which resulted in the analysis of a large collection of tested meningioma samples. We confirmed previous findings regarding meningioma biomarkers [3, 5, 9, 11, 12, 16, 19, 21, 28, 30, 32, 38, 39, 43–45, 48, 52, 56]. All investigated markers tested positive in all meningiomas, regardless of WHO grade. However, SSTR-2 expression was especially robust with a “high score” in 81.8% of all cases. SSTR-2 was also highly specific and sensitive. These findings are in line with previous studies [12, 30, 48]. Subsequently we focused on the second aim of this study, namely, identifying the most promising tumour-specific marker for intraoperative application. Applying TASC, SSTR-2 was found to be superior when compared to the other three biomarkers. Although TASC was initially developed for target selection in colorectal cancer and has not yet been validated in other tumours, the principle is also applicable to other tumour types as it is based on biomarker characteristics and not on a specific tumour type. This tool (TASC) is the first structured method to determine the suitability of a biomarker for intraoperative imaging. As a major advantage, TASC provides an objective score by considering available evidence.

The potential of SSTR-2 was analysed in a translational model using newly generated patient-derived 3D meningioma cultures. In two of three tested cultures, SSTR-2 expression was present at the cell membrane, emphasizing the possibility of SSTR-2 as a potential target for fluorescence guided imaging. One culture was SSTR-2 negative, which may be due to a technical issue with reduced culture viability. Indeed, the original patient tumour was SSTR-2 positive. Further research is warranted to investigate this issue further. As far as we know, this is the first time that the expression SSTR-2 has been determined in in vitro meningioma cultures using IHC. Several limitations became apparent when using this model. The cultures could be subcultured for a limited number of passages: cell growth decreased after three to four passages, similar to a previous report [51]. Moreover, the generated in vitro models are all originating from WHO grade I meningiomas. Although patients with high grade lesions may benefit the most from intraoperative imaging, WHO I meningiomas are still a representable model as SSTR-2 is expressed in all meningiomas, regardless the grade [30, 48].

The present study is an essential first step towards the development of meningioma-specific intraoperative fluorescence-guided imaging. Future steps should consist of binding studies with fluorescent dyes (preferably near-infrared dyes, such as IRDye 800CW) with SSTR-2 analogues (e.g. octreotate). These have already been used in targeted therapy for recurrent meningiomas [2, 8, 42] and their application in theranostics and PET-scanning has been demonstrated [19, 21, 52]. IRDye 800 CW has undergone a microdosing study [27] and has been applied in various clinical trials [57]. Further validation is needed by testing a target-directed imaging tool in vitro for proof of concept, and subsequently in in vivo animal models using xenograft mouse models. Animal models have been successfully applied previously, using fresh patient-derived material [20, 31, 41, 51] or immortalized meningioma cell lines [4, 7, 40, 41], with higher grade meningiomas yielding a higher success rate. Such models are needed for translational research to assess SSTR-2 guided intra-operative meningioma surgery.

## Conclusions

The present results highlight the potential of SSTR-2 as a high potential target for fluorescence-guided imaging. We identified SSTR-2 as the most suitable biomarker for targeted fluorescence-guided meningioma surgery by applying TMA-IHC and TASC. SSTR-2 was highly sensitive and specific and was expressed in all meningiomas in our large patient sample cohort, regardless of WHO grade. Furthermore, we established freshly generated in vitro meningioma models closely reflecting the original patient tumour and confirmed SSTR-2 expression in three meningioma cultures, which marks the first and essential step towards future in vitro experiments (tumour-cell imaging with fluorescently labelled SSTR-2 receptor markers) and in vivo experiments (meningioma identification in a mouse model). Further preclinical studies need to be performed to further develop the concept for targeted fluorescence-guided meningioma surgery.

## Abbreviations

EMA: Epithelial membrane antigen
IHC: Immunohistochemistry
PDGFR-β: Platelet-derived growth factor receptor beta
SSTR-2: Somatostatin receptor type 2
T/N: Tumour-to-normal tissue ratio
TASC: TArget Selection Criteria
TMA: Tissue microarray
VEGF-α: Vascular endothelial growth factor A
